# Biomaterials as carrier, barrier and reactor for cell-based regenerative medicine

**DOI:** 10.1007/s13238-015-0179-8

**Published:** 2015-06-19

**Authors:** Chunxiao Qi, Xiaojun Yan, Chenyu Huang, Alexander Melerzanov, Yanan Du

**Affiliations:** Department of Biomedical Engineering, School of Medicine, Tsinghua University, Beijing, 100084 China; Collaborative Innovation Center for Diagnosis and Treatment of Infectious Diseases, Hangzhou, 310003 China; Department of Plastic and Reconstructive Surgery, Beijing Tsinghua Changgung Hospital; Medical Center, Tsinghua University, Beijing, 102218 China; Cellular and Molecular Technologies Laboratory, MIPT, Dolgoprudny, 141701 Russia

**Keywords:** carrier, barrier, reactor, biomaterial-assisted therapy, regenerative medicine

## Abstract

Cell therapy has achieved tremendous success in regenerative medicine in the past several decades. However, challenges such as cell loss, death and immune-rejection after transplantation still persist. Biomaterials have been designed as carriers to deliver cells to desirable region for local tissue regeneration; as barriers to protect transplanted cells from host immune attack; or as reactors to stimulate host cell recruitment, homing and differentiation. With the assistance of biomaterials, improvement in treatment efficiency has been demonstrated in numerous animal models of degenerative diseases compared with routine free cell-based therapy. Emerging clinical applications of biomaterial assisted cell therapies further highlight their great promise in regenerative therapy and even cure for complex diseases, which have been failed to realize by conventional therapeutic approaches.

## Introduction

Cell-based therapy has emerged as a central strategy in regenerative medicine and tissue engineering. Over the past few decades, various cell types, including primary cells (Kulig and Vacanti, 2004; Pepper et al., 2015), stem cells (Street et al., 2003; Astradsson, 2015; Tomatsu et al., 2015; Barczyk et al., 2015), and genetically modified cells (Kim et al., 2001), have been chosen as potential candidates to treat a myriad of complex diseases (e.g. diabetes (Efrat, 2008; Pouch, 2015), anemia (Orive et al., 2005), hemophilia (Hortelano and Chang, 2000) and even cancers (Cirone et al., 2002; Hao et al., 2005)). A common drawback of cell therapy based on free cell transplantation is the loss of more than 90% cells within the first few hours after delivery, implying that only a small portion of the transplanted cells are eventually engrafted at tissues or organ sites of interest (Mooney and Vandenburgh, 2008; Hofmann et al., 2005; Fisher and Strom, 2006). Tissue engineering with the aim of replacing lost organ functions or promoting body’s natural repair process provide an alternative approach to treat complex diseases (Kearney and Mooney, 2013; Chan and Mooney, 2008). Such therapy depends on the interplay among biomaterials, cells, and growth factors to provide a proper microenvironment for treatment of diseased organs or tissue regeneration. Biomaterials play central role in tissue engineering for delivering cells or growth factors effectively. Biomaterials can function as carriers to deliver cells to desirable area and induce local tissue regeneration; as barriers to protect transplanted cells or tissues from host immune attack; or as reactors to stimulate host cell recruitment, homing, and differentiation (Kearney and Mooney, 2013). An ideal biomaterial designed for tissue engineering should be able to ensure high cell survival rate, appropriate cell function after transplantation and induce autologous functional tissue growth *in situ*, along with its own degeneration with the completion of treatment. Besides, comprehensive characterizations of biomaterials, varying in geometrical structure, physical form, chemical properties, and biocompatibility, should be assessed prior to their applications. In this review, functions of biomaterials as carriers, barriers, or reactors during cell-based regenerative medicine are discussed respectively to shed light on directions for future development of optimally functional biomaterials in regenerative medicine.

## Biomaterials as carriers

One reason for limited therapeutic efficiency of free cell transplantation is that many types of cells are anchorage-dependent and subjected to anoikis during or immediately after transplantation. Therefore, to improve therapeutic effectiveness, successful delivery of live and functional cells to lesion sites is crucial for subsequent regeneration. Biomaterials can be used as carriers to promote tissue regeneration and accelerate relatively large wound healing by delivering cells to injured sites and maintaining their viability. Currently, various biomaterials have been applied to promote tissue regeneration through different mechanisms by accommodating cells in three-dimensional (3D) microenvironment (Wang et al., 2010). As cell carrier, which is usually referred to as scaffold (Langer and Vacanti, 1993), biomaterials can provide anchoring ligands for cells, thereby providing proper microenvironment in which transplanted cells can survive better, migrate more effectively to desired sites and thus function more efficiently (Kim et al., 2000; Malafaya et al., 2007). In the following section, more detailed examples of transplantable (with surgery on host) and injectable (minimally invasive strategy) carriers used in regenerative medicine are reviewed.

### Transplantable carriers

Cell carriers such as decellularized organs, pre-gelated hydrogels, and other artificially fabricated scaffolds are generally transplanted in host via surgery due to the bulky size, which renders injection impossible. Both natural and synthetic polymers have been used as raw scaffold materials with innate cell binding sites or through surface modification during or after fabrication. Various cell types (e.g. hepatocytes, fibroblasts, and chondrocytes) derived from autologous or allogeneic origin have been seeded in these carriers, cultured *ex vivo*, and transplanted to liver Mooney et al. (1995), skin Cooper et al. (1991), and cartilage Cao et al. (1997). These cells could also be genetically modified *ex vivo* to overexpress and secrete specific factors. A combination of cells and growth factors could also be carried to the lesion site by the biomaterials.

#### Natural scaffolds as carriers

Natural extracellular matrix (ECM) produced by organ decellularization provides ideal carrier for cell transplantation, which retains almost intact vasculature system and complex architecture of the original organ. ECM is the major component of the naturally occurring cellular microenvironment, which is secreted and remodeled by the resident cells. Major components of ECM, regardless of tissue origin, includes proteins (e.g. collagen, laminin, fibronectin) and polysaccharides (e.g. hyaluronic acid) (He and Callanan, 2013). These components contain binding motifs which are specific peptide sequences interacting with integrin on cell membranes (Giancotti and Ruoslahti, 1999; Stupack and Cheresh, 2002). Recent studies revealed that ECM not only serves as substrate for cell attachment and migration, but also provides binding domain for growth factors, including fibroblast growth factor (FGF), vascular endothelial growth factor (VEGF), and hepatic growth factor (HGF) (Bashkin et al., 1989; Sahni et al., 1998; Li et al., 2010; Martino and Hubbell, 2010; Martino et al., 2011). Such characteristics make decellularized ECM suitable for providing appropriate biophysical and physiological milieu for loaded cells (He and Callanan, 2013). To date, various organs have been successfully decellularized, including heart (Bader et al., 1998; Booth et al., 2002; Kasimir et al., 2003), liver (Lin et al., 2004), urinary bladder (Rosario et al., 2008; Freytes et al., 2004; Gilbert et al., 2005), skin (Chen et al., 2004), lung (Price et al., 2010; Daly et al., 2012), tendon (Cartmell and Dunn, 2000), blood vessels (Conklin et al., 2002; Dahl et al., 2003; Uchimura et al., 2003), nerves (Hudson et al., 2004), skeletal muscle (Borschel, 2004), ligaments (Woods and Gratzer, 2005), and small intestinal submucosa (Badylak et al., 1995). Cells reseeded in the decellularized scaffolds survive in an environment with mimicry to that *in vivo*, thus recellularized ECM imitating natural organs could be a promising alternative for organ transplantation and regeneration. Successful trials have been established in animal disease models of bladder (Yoo et al., 1998), skin (Schechner et al. 2003), heart (Ott et al., 2008; Wainwright et al., 2010), and lung (Cortiella et al., 2010; Petersen et al., 2010).

**Figure 1:**
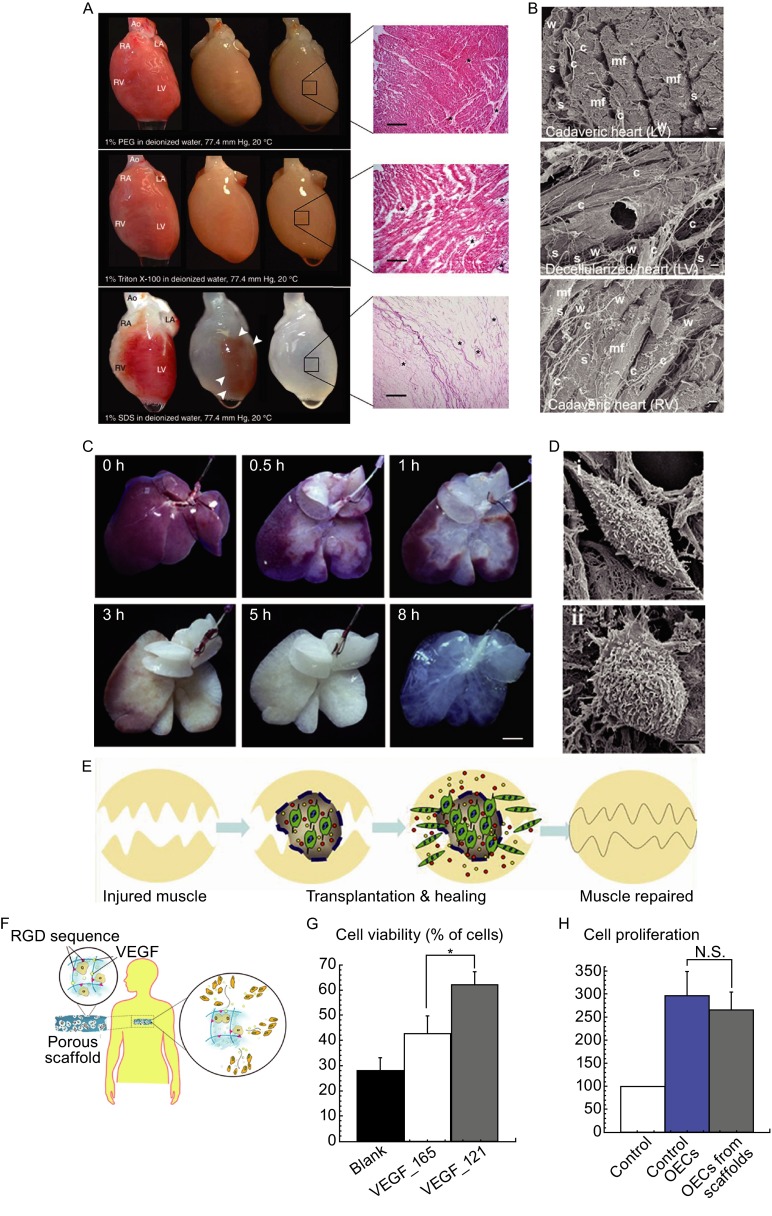
**Transplantable biomaterials as cell carriers**. (A) Perfusion-based decellularization of whole rat hearts and HE staining at different stages; (B) SEM of cadaveric and decellularized left ventricular (LV) and right ventricular (RV) myocardium, myofibers (mf), characteristic weaves (w), coils (c), struts (s), and dense epicardial fibers (epi) were retained (Ott et al., 2008); (C) General appearance of rat liver during decellularization process at different time points; (D) Ultrastructural characteristics of undifferentiated MSCs (i) and hepatocyte-like cells (ii) in biomatrix scaffold using SEM (Ji et al., 2012); (E) Engineered scaffold containing transplanted cells and growth factors is able to guide tissue regeneration *in situ* (Borselli et al., 2011); (F) Modification of RGD as morphogens on biomaterials providing cell adhesion ligands to maintain cell viability, and to activate and induce cell migration out of scaffold; (G) Viability of endothelial cells (OECs) that migrated out of scaffolds with no VEGF (blank), VEGF121 or VEGF165 in scaffolds; (H) Proliferation of cells that migrated out of scaffolds, normalized cell number (% of initial) (Silva et al., 2008). [Images are reproduced with the permission from Ott et al. (2008), Ji et al. (2012), Borselli et al. (2011), and Silva et al. (2008)]

Netoff’s group and Taylor’s group realized recellularization with cardiac or endothelial cells on cardiac ECM produced by decellularization via perfusion (Ott et al., 2008; Badylak et al., 2011). The biomimetic tissues could maintain functional contraction and be electrically stimulated *in vitro* for 28 days. Taylor’s group further optimized the cell seeding method to obtain more uniform distribution and transplanted the tissue into recipient rats (Badylak et al., 2011). Rats survived after the surgery and no immune reaction was observed until 7 days after transplantation, proving the functionality of the artificial heart *in vivo*. Similar research had been conducted in liver (Fig. 1A and 1B), where ECM with intact hepatic vasculature system was obtained by decellularization of liver. Ji et al. seeded mesenchymal stromal cells (MSCs) into the scaffold, cultured the artificial tissue *in vitro* in presence of growth factors to induce MSCs differentiation into hepatic lineage. The resulting tissue exhibited hepatic ultrastructure, which was transplanted into mice with liver failure induced by CCl_4_. The mice were rescued with liver regeneration thanks to paracrine factors of MSCs-differentiated hepatocytes (Ji et al., 2012) (Fig. 1C and 1D).

#### Synthetic scaffolds as carriers

Engineered scaffolds derived from both natural and synthetic polymers have been used as cell carriers as well. Cell binding sites are either modified on surface after scaffold formation, or naturally exist or supplemented into the scaffold during fabrication. Synthetic polymers, such as polylactide (PLA), polyglycolide (PGA), and their copolymer (PLGA), as well as hydroxyapatite, can be functionalized with serum proteins (e.g. fibronectin or vitronectin), to provide binding sites for cell adhesion (Chastain et al., 2006). Cells alone or with growth factors could be entrapped in such scaffolds, which are generally large in size, hence requiring surgery for transplantation.

As an example, genetically engineered MSCs derived from bone marrow, muscle, and adipose tissue with overexpressed bone morphogenetic protein (BMP) were delivered in scaffolds to bone defective models which demonstrated osteogenic potential (Kofron and Laurencin, 2006; Peterson et al., 2005; Sugiyama et al., 2005). Similarly, transfected cells with overexpressed VEGF have been delivered by scaffolds to promote angiogenesis, bone formation, as well as vasculature, in different animal models (Jabbarzadeh et al., 2008; Peng et al., 2002; Blumenthal et al., 2010). MSCs with intrinsic high expression of VEGF are also cell sources for transplantation to promote wound healing (Egana et al., 2009). In other situations, unmodified cells are delivered along with growth factors to enhance therapeutic efficiency. Such combinations have been realized for MSC delivery with rhBMP-2 in alginate for bone regeneration (Simmons et al., 2004), and with transforming growth factor β1 (TGF-β1) in gelatin scaffolds for cartilage regeneration (Park et al., 2007). Other than MSCs, cells derived from embryonic stem cells (ESCs) and induced pluripotent stem cells (iPSCs) have also been entrapped in biomaterials and transplanted for disease treatment (Klees et al., 2008; Elisseeff et al., 2006; Hwang et al., 2008). Meanwhile, terminally differentiated somatic cells such as endothelial cells, myoblasts, and fibroblasts, were transplanted to exert therapeutic function *in vivo* (Park et al., 2007; Koffler et al., 2011).

Ideally, transplanted cells can migrate out from the carriers into lesion areas to perform their function or regulate local regeneration via direct interaction with the host cells. In an example for skeletal muscle repair, alginate cryogel-based scaffold loaded with VEGF, insulin-like growth factor 1 (IGF-1) and myoblasts was transplanted into mice with hind limb ischemia. Controlled release of VEGF and IGF-1 and improved myoblast engraftment in injured skeletal muscle resulted in rapid regeneration and limited fibrosis (Borselli et al., 2011) (Fig. 1E). In another related work, VEGF along with endothelial progenitor cells were transplanted, and full limb perfusion was observed after 40 days of transplantation (Silva et al., 2008) (Fig. 1F).

### Injectable carriers

For patients with end stage diseases, invasive treatment or operation may be difficult to cope with. Besides, certain tissue, such as intervertebral disc, is not easily accessible for surgical repair without mechanical damage to its original structure. Therefore minimally invasive strategy to treat these special patients or organs/tissues is in high demand. Currently, one of the most widely used injectable carriers is hydrogel due to its unique characteristics including the ability to fit defective cavities of any shape and size, quick gelation and construction for uniform distribution of transplanted cells, and high water content to mimic native tissue (Wang et al., 2010). Injectable hydrogel can gelate *in situ* via ionic crosslinking or temperature change. Many successful pre-clinical and clinical trials including islet (Weber et al., 2007), cartilage (Elisseeff et al., 2000), liver (Tsang, 2007), cornea (McLaughlin et al., 2009), nerve (Cheng et al., 2013), and other organs have demonstrated the feasibility of hydrogel-based cell therapy. An inherent drawback for hydrogel, however, is the limited pore size in the polymeric network, which only allows for diffusion of medium, metabolites, and nutrients, but hinders migration of transplanted cells out of the carrier. This renders hydrogel as a better barrier than carrier. Despite this limitation, hydrogel is still an ideal carrier system when direct cell-cell interaction between transplanted cells and host cells is not crucial and transplanted cells function mainly via paracrine effects.

**Figure 2:**
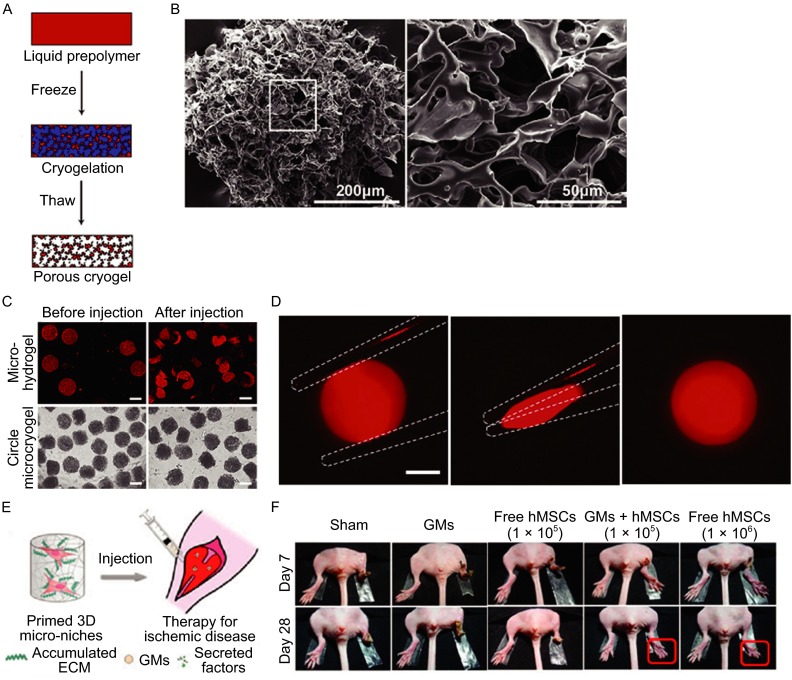
**Injectable cryogels for cell transplantation**. (A) Gelation process of cryogels; (B) SEM of highly porous PEG cryogels; (C) Microscopic images of microhydrogels (fluorescently stained by Nile red for enhanced visualization) and the microcryogels with different shapes before and after injection (scale bar = 500 μm); (D) Images demonstrating ability of an individual rhodamine-gelatin cryogel to be compressed between forceps (dashed *white line*) to large strain, followed by release and resumption of its original shape (Koshy et al., 2014); (E) Primed 3D microniches that can be injected into mouse hindlimb; (F) Representative photographs of sham, blank GMs (gelatin microniches), free hMSCs (10^5^), hMSCs (10^5^) within GMs (GMs + hMSCs), and free hMSCs (10^6^) at 7 and 28 days after treatment (Li et al., 2014). (Images are reproduced with the permission from Li et al. (2014) and Koshy et al. (2014))

As alternatives to hydrogel, scaffolds with relatively larger pores such as cryogel are suitable for cell loading, hence avoiding cell damage during gelation of hydrogel; and also providing sufficient space for cell proliferation (Li et al., 2014). The macro-porous cryogels are mechanically stronger scaffolds with pre-defined size and shape, which enable automatic and homogeneous cell loading and function as injectable cell delivery vehicles. Cells can be primed *in vitro* before transplantation into lesion area to facilitate long-term therapeutic effect, in which ECM accumulation and cell-cell interactions construct a favorable cellular microenvironment and therefore avoid immediate exposure of transplanted cells to ischemic and inflammatory environment *in vivo*. To realize this purpose, Koshy et al. developed an injectable, porous and cell-responsive gelatin cryogel that could withstand large strain from forceps compression, without obvious deformation, making injection easier to handle. Li et al. have developed poly-ethylene-glycol (PEG) and gelatin microcryogel systems (from 200–800 μm in diameter) that could be site-directed injected *in vivo* without significant damage to loaded fibroblasts or MSCs (Liu et al., 2014) (Fig. 2B and 2C). The cell carriers were applied to treat hind limb ischemia in mice. After priming the seeded MSCs *in vitro* for 2 days, the 3D microscale cellular niches were deposited with ECM essential for cell survival. The microcryogels also protected the cells from mechanical damage during injection and provided cell retention *in vivo*. Ultimately, only one-tenth of cells compared to that used in conventional free cell therapy were required to achieve even better treatment outcomes as shown by fluorescent imaging of blood perfusion in ischemic hind limb (Li et al., 2014) (Fig. 2E and 2F).

## Biomaterials as barriers

While cases shown above depict the success of biomaterial-assisted cell delivery to lesion sites for improved therapy, another big challenge is the immune rejection of transplanted allo- or xeno-geneic cells. If cells are transplanted without protection, host immune system would recognize these foreign antigens quickly and immediate rejection of cellular grafts occurs. Hydrogels are mainly used to encapsulate cells for immune-isolation, which are either biodegradable for short-term application (e.g. oral delivery of genetically engineered *E. coli* to remove urea (Prakash and Chang, 1996)) or mechanically stable materials for treatment of chronic diseases (e.g. diabetes). The highly hydrated microenvironment of hydrogels enabled embedded cells to be instructed to differentiate, proliferate, and migrate (Vermonden et al., 2008).

### Mechanism of immunoisolation

**Figure 3:**
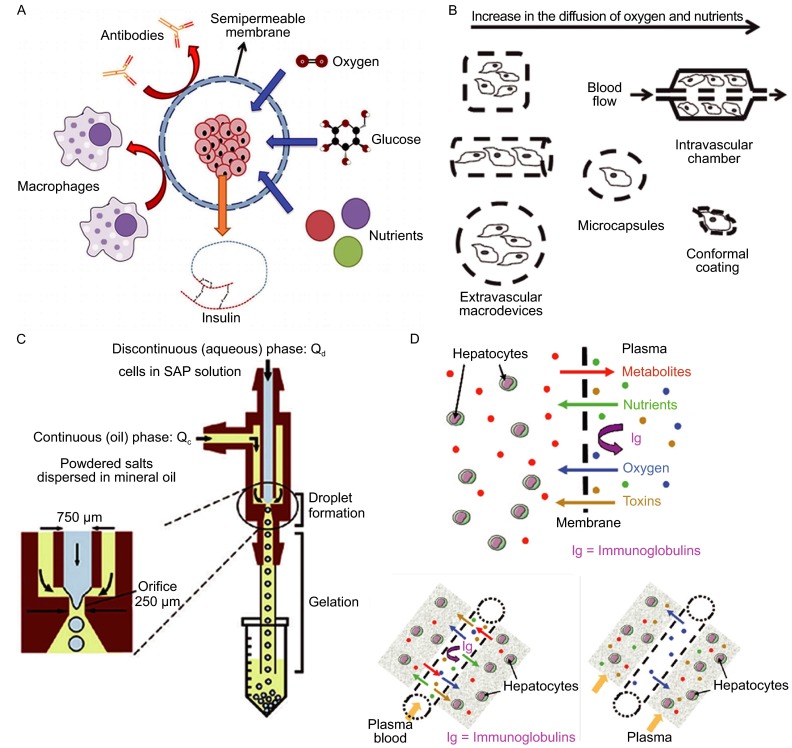
**Immunoisolation principles based on biomaterials and devices currently used as barriers**. (A) Principle of immunoisolation by a microcapsule. The semipermeable membrane allows diffusion of molecules such as nutrients, glucose, oxygen, and insulin, and protects the graft from effector molecules of host immune system (Juarez, 2014); (B) Conventional and novel encapsulation devices used for cell immunoisolation (Nafea et al., 2011); (C) A monodispersed cell-laden microbead fabrication method by using axisymmetric flow-focusing devices (Kang et al., 2014); (D) Principle of bioartificial livers. Plasma transfers nutrients and oxygen to hepatocyte-housing compartment across membrane to maintain cell viability. Toxins in plasma are eliminated by hepatocytes via hepatic metabolism. Metabolic substances are returned to blood steam; (E and F) Exchange principles between hepatocytes and plasma or blood in the two types of bioartifical liver devices (Carpentier et al. 2009). (Images are reproduced with the permission from Juarez (2014), Nafea et al. (2011), Carpentier et al. (2009) and Kang et al. (2014))

Immune rejection is primarily due to hyperacute rejection (HAR) where host antibodies target antigens on the surfaces of the transplanted cells (Krishnamurthy and Gimi, 2011), and sequentially activate immune response to eliminate those cells. Without survival at the lesion site, transplanted cells cannot exert therapeutic functions. With respect to this, transplanted cells should be protected from surrounding environment to hinder host immune system’s accessibility to engrafts. Clinically, immunosuppressant is commonly applied to prevent immune rejection of transplanted organs or cells in patients, but long-term usage can render recipients vulnerable to infection, as well as susceptible to tumorigenesis (Hernandez et al., 2010). Alternatively, cells can be encapsulated within a semipermeable polymeric membrane to eliminate HAR by preventing cell-host contact (van der Windt et al., 2007). The semipermeable membrane physically permits bi-directional diffusion of small molecules (e.g. oxygen, carbon dioxide, cellular nutrients and growth factors, cellular waste products, ions, and therapeutic molecules secreted by entrapped cells) between host and transplants, while isolates encapsulated cells from host immune cells (e.g. neutrophils and macrophages), and prevents recognition of transplanted cells as foreign objects by antibodies and complements of the recipient’s immune system (Juarez, 2014) (Fig. 3A). Therefore, it is not necessary for recipients to use immunosuppressant, thus eliminating the severe side effects and undesired complications (Orive et al., 2003; Hunt and Grover, 2010). Materials providing protection to cells are desirable in immunoisolated therapy, in which xenograft cells or tissues are encapsulated and isolated from host immune system to ensure cell survival and clinical outcomes. To serve this purpose, non-adhesive microporous scaffolds or membranes fabricated from naturally derived polymers (e.g. alginate (De Vos et al., 1997; Omer et al., 2005; Lacy et al., 1991), and agarose (Schneider et al., 2001; Wong and Chang, 1991)) are desirable. These biomaterials are designed to isolate surrounding tissues, thereby making transplanted cells inaccessible to host immune system and increasing the probability of xenograft survival. By enclosing a transplant with a semipermeable barrier, an ‘artificial immunoprivileged site’ could be created to shield engraft from destruction of host immune system (Paul et al., 2009; Antosiak-Iwanska et al., 2009). Such protective strategy for cells/tissues transplantation has been demonstrated efficient in pathological reversal of many diseases, such as central nervous system diseases, diabetes mellitus, hepatic diseases, amyotrophic lateral sclerosis, hemophilia, hypothyroidism, and cardiovascular diseases (Zhang et al., 2008; Grandoso et al., 2007; Colton, 1995; Desai et al., 2000; Sellitto et al., 1995). As one of the excellent examples of biomaterials functioning as barrier, porcine islets encapsulated in non-degradable alginate were delivered into small or large non-human primates with diabetes to maintain normoglycaemia for as long as 2.4 years (O’Sullivan et al., 2011).

### Intracorporeal barriers

Encapsulation techniques are typically classified as macrocapsulation (usually as flat-sheet or hollow-core fibers) and microencapsulation (usually as small spherical vehicles or conformally coated cells/aggregates). Whichever technique used, the basic principle is to produce cell-laden droplets with controlled size, followed by stabilization of the droplets via interfacial processing and subsequent generation of solid capsule membrane surrounding the droplets. A central principle for choosing biomaterials for encapsulation is that crosslinking agents should be non-cytotoxic before and after gelation. Immunoisolation devices used in pre- and clinical trials can be classified as intracorporeal cell/tissue transplantation (e.g. islets transplantation) and extracorporeal functional assisted devices (e.g. bioartifical liver).

#### Micro-encapsulation strategies

Microcapsules, characterized by dimensions in the order of hundreds of microns or less, are spherical in shape to take advantage of the optimal surface-to-volume ratio for improved protein and nutrient diffusion which can maintain good cell viability. The microscale size makes microcapsule ideal barriers for transplantation into microvascularized and deep tissue sites. Natural polymers derived from non-animal sources or synthetic polymers that do not contain cell binding sites are superior materials for use as barriers. Cells are generally encapsulated within nanoporous biodegradable hydrogels, such as hyaluronic acid, fibrin, or gelatin, or nondegradable hydrogels such as alginate, or PEG, which regulate cross-membrane diffusion of nutrients, oxygen, waste and therapeutic agents produced by the encapsulated cells (Nicodemus and Bryant, 2008). With great success in short or intermediate term cell therapy, alginate is the most commonly used polymer matrix to generate cell-laden microcapsules (Zimmermann et al., 2007). Typically, cells are suspended in alginate pre-solution, which is extruded through a droplet generator into a calcium chloride bath, leading to formation of microcapsules (Fig. 3C). Efforts have been made to improve alginate’s mechanical stability, such as layering poly-L-lysine (Lim and Sun, 1980), poly-l-orinthine 9 (De Vos et al., 1993), and poly(methylene-co-guanide) on surface of the microcapsules (Calafiore et al., 1999; Wang et al., 1997), and control homogeneity of its porosity using multilayered poly-electrolytes (Krol et al., 2006; Weber et al., 2008).

Another method to form microencapsulation is conformal coating of cells, where a thin coating covered on engrafted cells or cell aggregates could reduce diffusion rate of molecular between transplants and host, hence a prolonged graft response to host is achieved (Krishnamurthy and Gimi, 2011). Layer-by-layer coating of poly-electrolytes enables precise control of nanometer thickness of the coating around the engraft. For example, Wyman et al. conformally coated pancreatic islets with an oil-water bilayer where high density chlorinated hydrocarbon oil was used. Islets floated at the interface in-between water and oil, since islets were heavier than water but lighter than oil. When oil was withdrawn, islets would be entrained in a thin layer of oil suspended on the aqueous layer. The aqueous layer contained photo-polymerizable PEG-diacrylate mixed with eosin that worked as photo-initiator, which was then crosslinked to form a uniform PEG layer about 50 μm thick outside the islets (Wyman et al., 2007).

Though poly-electrolytes have been widely used in conformal coating, lack of well-controlled porous properties still limit their application in short and intermediate term cell therapy, because any failure leading to host antibody transmit would damage the immunoprotective function of the entrapped cells. Thus nanofabricated membranes with uniform and reproducible porosity are preferable for immunoisolation, especially in long-term application. Various nanoporous membranes, with pore sizes ranging from 10 nm to 55 nm, have been developed to improve microencapsulation (Desai et al., 1999; Kumar et al., 1999; d Graaff et al., 2003) (Fig. 3B). However, simply reducing pore size to block antibodies, especially IgG seems to be in conflict with the required sufficient perfusion of essential growth factors; hence strategies for modifying nano-pores with capacity to deactivate immunoglobulin may be important design factors for consideration (Dionne et al., 1996; Iwata et al., 1995).

#### Macro-encapsulation strategies

For macro-encapsulation, capsules with dimensions in the order of 0.5–1.5 mm in diameter and several centimeters in length are common. With increase in volume, more cells can be loaded, which saves on the number of capsules correspondingly. Capsules for macro-encapsulation are commonly covered with thick membrane that is mechanically stable but at the same time limits nutrient/waste diffusion. Immobilizing the capsules around vasculatures can ease the mass-transfer problem, but close contact with circulating blood will subsequently induce enhanced response from the host. Intravascular devices for nutrient supply to the macro-encapsulated transplants were applied in early days, but were gradually replaced due to thrombosis after surgery (de Vos and Marchetti, 2002). Extravascular devices, classified as extravascular macrodevices and extravascular microcapsules, rely on surrounding blood vessels (Uludag et al., 2000) and avoid thrombosis risk. Extravascular macrodevices are designed as macrocapsules, planner membranes or hollow fibers, all of which have the advantages of encapsulating large number of cells and the versatility of cell retrieval from implantation site in case of post-operative complications. Due to the small surface-to-volume ratio and inefficient oxygen and nutrient diffusion, cell necrosis usually manifests. Besides, these devices have been reported to yield poor mechanical property and biocompatibility (Nafea et al., 2011). Extravascular microcapsules, on the other hand, generally refer to devices with diameters less than 1 mm with small number of cells encapsulated. Such devices circumvent drawbacks of extravascular macrodevices, but significantly lower the possibility of retrieval after usage (Kizilel et al., 2005).

### Extracorporeal barriers

Extracorporeal function-assisted devices, such as artificial liver and pancreas, are large medical systems that provide short-term assistance in function compensation of a particular organ. They facilitate regular exchange and supplement of bioactive factors for cell functional maintenance. The extracorporeal devices are usually connected to recipients’ circulation system, which transmit functional factors into the host blood stream without direct interaction. There are two types of extracorporeal devices for temporary support: artificial and bioartificial support devices. Artificial support systems essentially use non-living components to remove the toxins accumulated in blood stream. Bioartificial support devices incorporate biologically living components (e.g. hepatocytes or islets) to provide biotransformative or synthetic functions. For example, artificial liver devices essentially use non-living components to remove the toxins accumulated due to liver failure, and several systems have been approved by Food and Drug Administration (FDA) and are Council of Europe (CE) labeled (e.g. conforms with health and safety standards of the European Union). Meanwhile, bio-artificial liver devices contain a cell-housing bioreactor, whose role is to replace the primary liver functions (i.e. oxidative detoxification, biotransformation, excretion, and synthesis) (Carpentier et al., 2009). Four types of bioartificial liver devices are currently under investigation, which are either based on hollow fiber cartridges or chambers (i.e. ELAD, HepatAssist, MELS), monolayer cultures, or perfused matrices (i.e. BLSS, AMC-BAL) (David et al., 2004; Khalil et al., 2001) (Fig. 3D–F).

All the above mentioned efforts were made to direct the fate of transplanted cells or host cells that take part in tissue remodeling and rebuilding on a basic recognition that immune system was regarded as a negative regulator of cell functionalities. However, recent trials shown that acute immune response partially accelerate tissue regeneration via active modulation, such as promotion of vascularization, as tested in a mouse model (Kyriakides et al., 1999). While a chronic foreign body response should be avoided to prevent impedance of tissue regeneration by inflammation and fibrosis (characteristics of chronic foreign body response), it is suggested that modulation rather than avoidance of immune response is more desirable for tissue remodeling, which should be taken into consideration for developing immunoisolation strategies.

## Biomaterials as reactors

Classic biomaterials (e.g. long-lasting metals, ceramics, and polymer composites) have been successfully applied in clinic to compensate for loss of mechanical functions in injured tissues such as teeth, hips, knees, heart valves, and intervertebral discs. But they rarely modulate the host to repair and regenerate neotissues (Chan and Mooney, 2008). The limitation motivated the development of functional biomaterials capable of stimulating the innate regenerative capacity of the treated tissues (Balasundaram and Webster, 2007). When sufficient cells exist endogenously for repairment or regeneration, biomaterials can play an inductive role by attracting these endogenous cells and directing them to commit to differentiation and regeneration. Stem cells and progenitor cells with tremendous proliferative and regenerative capacity are thus the main focus of research for cell recruitment and induction by biomaterials *in vivo*. Biomaterials provide a framework for cell attachment, ECM deposition and subsequent differentiation into a designated lineage (Lutolf et al., 2009). Ideally, the template scaffolds could degenerate accompanied by the invasion and proliferation of host cells. As a result, new tissue is formed in absence of xenograft and function normally as the native counterpart.

### Growth factor-free biomaterials as reactors

**Figure 4:**
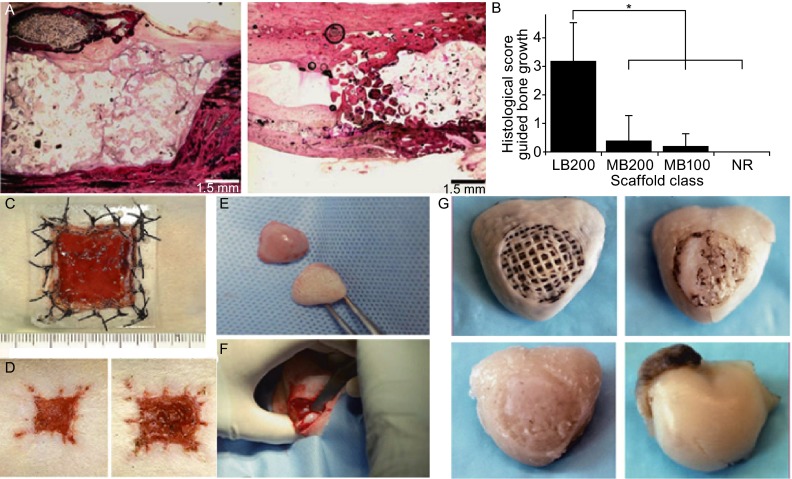
**Biomaterials applied as reactors for bone, skin, and cartilage regeneration**. (A) Histological sections of PPF/PLGA scaffolds, polyester poly(propylene fumarate) (PPF). Left, bone grown into and around PPF/PLGA scaffold. Right, bone did not grow into scaffold but grow along the external surface of the scaffold; (B) Histological scoring of longitudinal sections for bone growth around the outside of the implant (guided growth) in different scaffolds (Hedberg et al., 2005); (C and D) SMA-FP reduced *in vivo* wound contraction, smooth muscle actin (SMA); fusion peptide (FP) (Hinz et al. 2002). (D) Left, SMA-FP transplantation. Right, FP transplantation; (E–G) TGFβ3-collagen hydrogel promoted regeneration of the rabbit synovial joint. (E) Photograph of artificial and native synovial joint; (F) Surgical transplantation; (G) (i) Bio-scaffold prior to implantation, (ii) TGFβ3-free, (iii) TGFβ3-infused bio-scaffold after implantation for 4 months, and (iv) native cartilage; (H–K) TGFβ1 coated poly(caprolactone) (PCL) scaffold recruited mesenchymal cells for chondrogenesis (Lee et al. 2010). (Images are reproduced with the permission from Hinz et al. (2002), Hedberg et al. (2005) and Lee et al. (2010))

Scaffolds derived from purified ECM components (e.g. collagen, hyaluronic acid (HA), and fibrin) can be potentially less immunogenic with similar biochemical and structural moieties to natural ECM (Matthews et al., 2002). Promising therapeutic results have been shown by purified ECM component in tissue repair (Hubbell, 2003). Collagen is among the most widely used biomaterials in this category, and can be derived from animal tissues (e.g. skin and tendon), as well as human tissues (e.g. placenta). It can be reconstituted into solid gels via pH or temperature alterations. Cell migration can occur in collagen scaffold with a relatively large mesh size (e.g. collagen sponge) (Wolf et al., 2003) or through matrix degradation by MMPs (Hinz et al., 2002). Collagen scaffolds have been used clinically for bone (Uludag et al., 2000) and cartilage (Okamoto et al., 2003) repair (Fig. 4). Combinational use of chondroitin sulfate and collagen have been applied in skin (Butler et al., 1999) and peripheral nerves (Chamberlain et al., 2000) repair. Fibrin, a specialized ECM protein that participates in spontaneous tissue repair, has been applied in sutureless fixation of skin grafts (Currie et al., 2001). Other than natural ECM components, synthetic materials have also been applied. In a successful experiment of bone-tissue engineering, biodegradable polyurethane scaffold was transplanted to non-union fractures, which recruited MSCs and osteo-progenitor cells to heal the wound (Brown et al., 2011).

### Growth factor-loaded biomaterials as reactors

With the advance in research on biochemical factors that control and direct cell migration, differentiation, and proliferation, biomaterials designed to incorporate and enable controlled release of bioactive factors after transplantation provide local induction of cellular behaviors (Kearney and Mooney, 2013). Cells respond to a variety of stimuli present in the ECM, which compose of fibrous proteins, proteoglycans, and glycoproteins, and act as a main regulatory and structural component of tissues *in vivo*. Biomaterials incorporating bioactive factors (e.g. growth factors) have been extensively investigated in tissue engineering (Lutolf and Hubbell, 2005). In order to recruit endogenous cells for enhanced tissue regeneration and repair, growth factors, such as TGF-β, fibroblast growth factor (FGF), VEGF, epidermal growth factor (EGF), and platelet derived growth factor (PDGF), have been further modified to be controlled release from biomaterials (Metcalfe and Ferguson, 2007). For biomaterials lacking cell and growth factor binding sites (e.g. alginate, poly(bis(pcarboxy) methane anhydride), poly(propylene fumarate)), RGD peptides have been supplemented to incorporate growth factors such as rhBMP-2 (Kolambkar et al., 2011) and osteogenic thrombin peptide (Hedberg et al., 2005).

Since decellularized ECM could maintain relevant intact structures and biochemical compositions of natural tissue, they provide ideal reactors to facilitate tissue regeneration. Several decellularized ECMs have been commercialized to repair soft tissues, such as the FDA approved GraftJacket® (ECM derived from human dermis by Wright Medical Technology (Valentin et al., 2006)) and Medeor® Matrix (ECM derived from porcine dermis). However, immunogenicity, disease transmission, and wide variability are all potential drawbacks for decellularized ECM products to be applied as a reliable therapeutic device.

Due to tremendous proliferative and regenerative capacity, stem cells and progenitor cells, which reside in synovium, bone marrow, adipose, and vasculature, are expected to be the main cell types recruited and induced by biomaterials *in vivo*. For instance, MSCs have been recruited for regenerating defect cartilage (Fig. 4E–G). Since TGF-β is known with the ability to recruit MSCs and stimulate their chondrogenic differentiation (Noth et al., 2008), biomaterials (e.g. collagen and polycaprolacton) enabling control release of TGF-β have been used to generate cartilage in different animal models and patients (Noth et al., 2008; Gille et al., 2010; Lee et al., 2010; Huang et al., 2002). In a clinical trial, a complex composed of fibrin and collagen for controlled release of TGF-β was transplanted into patient with focal cartilage defects in the knee, which were completely filled in a 2 years follow-up study (Gille et al., 2010).

Other than stem cells and progenitor cells, adult cells (e.g. fibroblast and smooth muscle cells) could also be preferentially enriched to the damage sites (e.g. burns, trauma, surgery or diabetic foot ulcers) via growth factors, such as PDGF, FGF, VEGF, and EGF (Lynch et al., 1987). For example, rhPDGF was entrapped in methylcellulose gel and transplanted into patient with diabetic foot ulcers, which could stimulate fibroblast recruitment and ECM deposition (Lynch et al., 1987). In another example, Ayvazyan et al. impregnated collagen-gelatin scaffold with bFGF to promote palatal mucosa wound healing in dogs (Ayvazyan et al., 2011). Two weeks after transplantation, the number and area of newly formed capillaries were significantly higher in the group treated with bFGF-loaded scaffolds than blank control. For hind limb ischemia, vascularization is vital to maintain normal blood supply. Thymosin beta 4 (CCSS-eTβ4), an angiogenic factor, was reported to promote cutaneous wound healing, which was entrapped into collagen-chitosan sponge scaffold and then transplanted into rats with hind limb ischemia. Twelve days after transplantation, significant increases in CD31-positive endothelial cells was observed (Ti et al., 2014). In other work for treatment of excisional wound, Greenhalgh (2013) and Liem et al. (2013) modified collagen scaffold with low concentration of nicotine at wound healing sites produced by artificial dermis at mouse back skin. Fourteen days later, neoepithelium length in nicotine transplantation group was significantly increased compared to that in nicotine-free groups, which indicated recruitment of endothelial cells. Pro-angiogenesis can also be a treatment strategy for cardiovascular disease (CVD). For example, biomaterials coated with cyclic RGD peptides or CD34 antibodies, which recognize circulating endothelial progenitor cells (EPCs), were transplanted into CVD porcine models to act as a pro-homing substrate for *in situ* EPCs capture from blood streams. The captured EPCs could efficiently proliferate and maintain proper haemostasis to minimize the risk of restenosis (Avci-Adali et al., 2008).

## Clinical applications of biomaterial-assisted regenerative medicine

In spite of tremendous progress in basic research on regenerative medicine over the past decades, there are limited clinically approved therapeutics based on tissue engineering principles (Webber et al., 2015). One concern raised in translational medicine is to what extent these laboratory studies on theoretical modeling, *in vitro* characterization or *in vivo* assessment based on animal models, can be predictive of performance and therapeutic efficacy in human (Meijer et al., 2008; Agata et al., 2010). Some of the greatest advances in applying regenerative medicine to the clinic are exemplified in the area of skin and bone regeneration as well as diabetes treatment by using fibroblasts, MSC, and islets respectively (Yamada et al., 2004; Marston et al., 2003). Biomaterials applied in these clinical trials play a multifaceted role by functioning as carrier, barrier or reactor. The following part will introduce clinical applications and ongoing clinical trials of biomaterial-assisted regenerative medicine for skin regeneration, bone regeneration and diabetes treatment.

Epicel® (commercialized by Genzyme) is among the earliest cell-based skin regenerative products used in clinic, which comprises thin autologous keratinocytes sheets (2–3 cell layers thick) cultured on a xenogeneic mouse feeder layer. A layer of irradiated immortal mouse fibroblasts provides autologous cells of patients with support matrix for cell attachment and growth (Bello et al., 2001). In another product for skin regeneration, Dermagraft® (commercialized by Organogenesis, Inc), neonatal fibroblasts are seeded onto a bioabsorbable polyglactin scaffold which showed great promise in healing of diabetic foot ulcers (Marston et al., 2003). An alternative FDA-approved product for treating both venous leg ulcers and diabetic foot ulcers is Apligraf® (commercialized by Organogenesis, Inc). It consists of a two-layered construct with a layer of neonatal karitinocytes seeded on a second layer of collagen matrix containing neonatal fibroblasts (Fishman et al., 2013). To facilitate the manufacture, standardization, storage, and transportation as well as regulatory approval, off-the-shelf acellular scaffold-based regenerative strategy show their advantages as exemplified by INTEGRA® Dermal Regeneration Template (commercialized by Integra Life Sciences, Inc). This scaffold has two layers: a lay of cross-linked matrix consisting of bovine type-1 collagen and another layer of silicone coated with shark chondroitin-6-sulfate. The collagen layer is intended for endogenous cells recruitment to regenerate functional tissue; and the silicone layer is designed to mimic a synthetic dermis to prevent the wound bed from infection, while reducing heat and moisture loss at the same time (Webber et al., 2015).

A clinical study in bone tissue engineering published in 2004 (Yamada et al., 2004) showed improved efficiency of tissue-engineered bone regeneration using MSCs and platelet-rich plasma (PRP). The authors first confirmed feasibility of the treatment on a dog mandible model before translating the tissue-engineered bone into clinical application, during which three patients with onlay plasty in the posterior maxilla or mandible were transplanted with bone grafts and showed good plasticity several months later. To further confirm bone regeneration after engrafts transplantation, another study was conducted by Asahinaet et al. (Kagami et al., 2011). In this study, autologous BMSCs together with scaffolds comprising of platelet-rich plasma gel and beta-tricalcium phosphate (β-TCP) were transplanted into patients with severe atrophy of alveolar bone. A 2-year observation showed bone regeneration in all patients, though significant variations between individual were observed. No side effect or related complication was reported, which may imply the relative safety of alveolar bone tissue engineering with the use of autologous BMSCs.

As for type 1 diabetes treatment, there have been five phase I/II clinical trials registered at ClinicalTrials.gov to date, all of which are conducting around the world via encapsulated allogeneic islet transplantation (Yang and Yoon, 2015). In one study sponsored by Novocell, 12 diabetic patients were enrolled in phase I/II clinical trials in USA and subjected to PEG-encapsulated islets transplantation subcutaneously. Meanwhile, Academisch Ziekenhuis van de Vrije Universiteit, Brussels, sponsored two registered phase II clinical trials in Belgium, both of which are currently recruiting volunteers. One of the trials was designed to transplant alginate encapsulated human islets intraperitoneally, and the other one was to explore potential implantation sites (i.e. peritoneum, omentum, and brachioradialis muscles), for encapsulated islet transplantation. The fourth clinical trial was registered by Beta-O2 Technologies in Sweden which was designed to explore the safety and efficacy of macroencapsulated human islet transplantation using bioartificial pancreas. The fifth phase I clinical trial launched by Cliniques in Belgium was reported but with no substantial result so far. Besides registered clinical trials, there was also a report on nonregistered clinical trial sponsored by Living Cell Technologies in Russia in 2007 on neonatal insulin-producing porcine pancreatic islets encapsulated within alginate/poly-L-ornithine/alginate (commercially known as DIABECELL®) (Dufrane and Gianello, 2012). Seven insulin-dependent diabetic patients received between one to three implants of DIABECELL® (5000 and 10,000 IEQ/kg). None showed marked adverse events until 96 weeks after transplantation and even two of them became insulin independent for up to 32 weeks (Scharp and Marchetti, 2014). Following this success, another three clinical trials were launched in New Zealand and Argentina, all of which were sponsored by Living Cell Technologies, but were similarly without official registration thus far (Yang and Yoon, 2015).

In addition to demonstrating efficacy on clinical trials as technology advances, clinical translation of cell-based regenerative therapy must also obtain approval from regulatory agency for safety with acceptable risk of side-effects. Safety concerns such as teratoma formation, immunogenicity, eventual form and tissue sites and biocompatibility of functional materials usually hinder the entire translational procedure and hence require special attentions. Taking the interspecies variability into consideration, immune system in rodents could not fully reproduce the immune response to an implanted construct in human, due to the differences between mouse and human immunology. Emphasis on the safety issue, though not always the first consideration for developing new technologies in laboratory, is nevertheless critical when developing new therapies aimed at clinical applications (Webber et al., 2015). Further efforts are expected in answer to the challenges in front of translational medicine, to develop time- and cost-intensive processes for the widespread clinical applications. Making guidelines and consensus for transplantation of biomaterial-assisted engraft via allogeneic, xenogeneic or autologous stem cell-derived source is another issue in translating cell-based therapy. Ongoing clinical studies are expected to reveal the safety and efficacy of biomaterial-assisted regenerative medicine in the next few years, although much more effort is required for the ultimate clinical translation.

## Conclusion and future perspective

Great advancements have been made in biomaterial assisted regenerative medicine in the past two decades, and the number of patients benefiting from this promising therapeutic strategy has also been increased. Multifunctional roles of biomaterials to improve cell retention, survival, and functionality during cell therapy are systematically reviewed here, mainly from three aspects: (1) biomaterials as cell carriers for efficient and targeted cell delivery to therapeutic sites; (2) biomaterials as semipermeable barriers to protect transplanted cells from host immune system; and (3) biomaterials as cell reactors to activate and recruit host cells for regeneration. Recent progress, clinical applications and emerging trends in these three aspects have also been summarized, which highlight great potentials of future biomaterial development with integration of multi-functionalities. Despite the tremendous improvement in efficiency and efficacy of biomaterials-assisted regenerative medicine, underlying therapeutic mechanisms have not been clearly understood, which renders safety a main concern for large-scale clinical application. Future endeavors can develop injectable biomaterial assisted cell therapy for minimally invasive treatment, and minimize cell damage during the entire procedure (e.g. gelation, injection, and retention), hence fully realize the synergistic effects of biomaterials for cell delivery, protection, and induction. We believe that deeper explorations in the mechanism will potentiate further development in regenerative medicine.

## Abbreviation

3D: three-dimensional
BMP: bone morphogenetic protein
CE: Council of Europe
CVD: cardiovascular disease
ECM: extracellular matrix
EGF: epidermal growth factor
EPCs: endothelial progenitor cells
ESCs: embryonic stem cells
FDA: Food and Drug administration
FGF: fibroblast growth factor
HGF: hepatic growth factor
HAR: hyperacute rejection
IgA: immunoglobulin A
IgG: immunoglobulin G
IgM: immunoglobulin M
iPSCs: induced pluripotent stem cells
MSCs: mesenchymal stromal cells
PDGF: platelet derived growth factor
PEG: poly-ethylene-glycol
PGA: polyglycolide
PLA: polylactide
PPB: poly(L-lysine)-g-poly(ethylene glycol)(biotin)
SA: streptavidin
TGF-β1: transforming growth factor β1
VEGF: vascular endothelial growth factor
